# Publisher Correction: Online photochemical derivatization enables comprehensive mass spectrometric analysis of unsaturated phospholipid isomers

**DOI:** 10.1038/s41467-019-08907-6

**Published:** 2019-02-19

**Authors:** Wenpeng Zhang, Donghui Zhang, Qinhua Chen, Junhan Wu, Zheng Ouyang, Yu Xia

**Affiliations:** 1 MOE Key Laboratory of Bioorganic Phosphorus Chemistry & Chemical Biology, Department of Chemistry and State Key Laboratory of Precision Measurement Technology and Instruments, Department of Precision Instrument, Tsinghua University, Beijing 100084, China; 2 Department of Chemistry, Purdue University, West Lafayette, Indiana 47907, USA; 3 Affiliated Dongfeng Hospital, Hubei University of Medicine, Shiyan, Hubei Province 442000 China

Correction to: *Nature Communications*; 10.1038/s41467-018-07963-8: published online 08 January 2019

The original version of this Article contained an error in Fig. 2f, in which the phospholipid was incorrectly labelled ‘PE’ rather than the correct ‘PC’. This has now been corrected in the PDF and HTML versions of the Article.

