# Supplementary material for: Online photochemical derivatization enables comprehensive mass spectrometric analysis of unsaturated phospholipid isomers
Source: Nat Commun. 2019 Jan 8;10:79. doi: 10.1038/s41467-018-07963-8 (PMC6325166; doi:10.1038/s41467-018-07963-8)
Supplement: Supplementary file 1 — Supplementary Information [file 41467_2018_7963_MOESM1_ESM.docx]

Supplementary Information for

**Online photochemical derivatization enables comprehensive mass spectrometric analysis of unsaturated phospholipid isomers**

Zhang *et al.*

Includes: Supplementary Figures 1-27

Supplementary Note 1

**
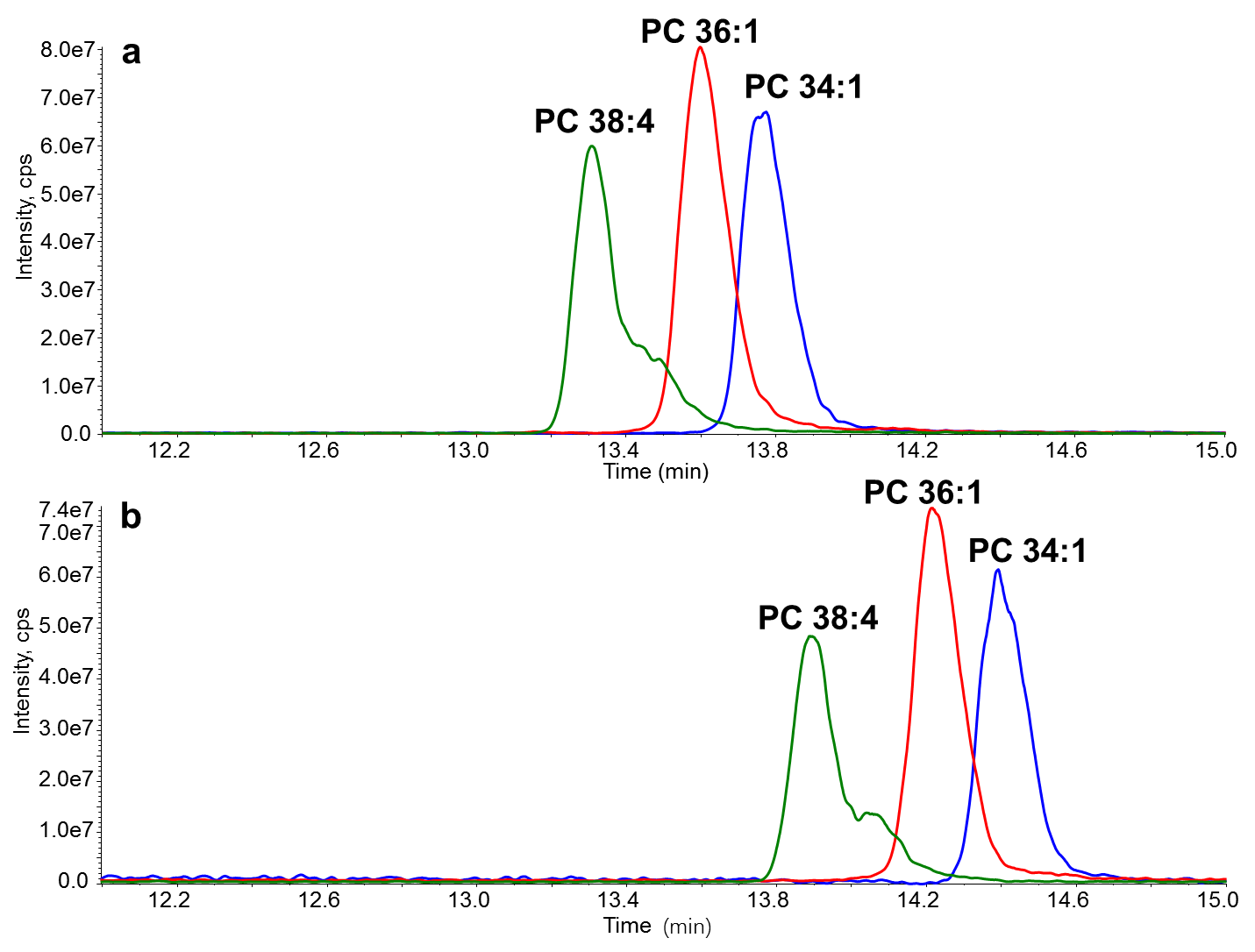
**

**Supplementary Figure 1.** Extracted ion chromatograms of PC (polar extract from bovine liver) on HILIC-LC-MS using different mobile phase systems: a) ACN/CH_3_COONH_4_ (ACN from 90% to 70% in 20 min) and acetone/ACN/ CH_3_COONH_4_ (acetonitrile/acetone, 50/50, v/v, from 90% to %70 in 20 min).


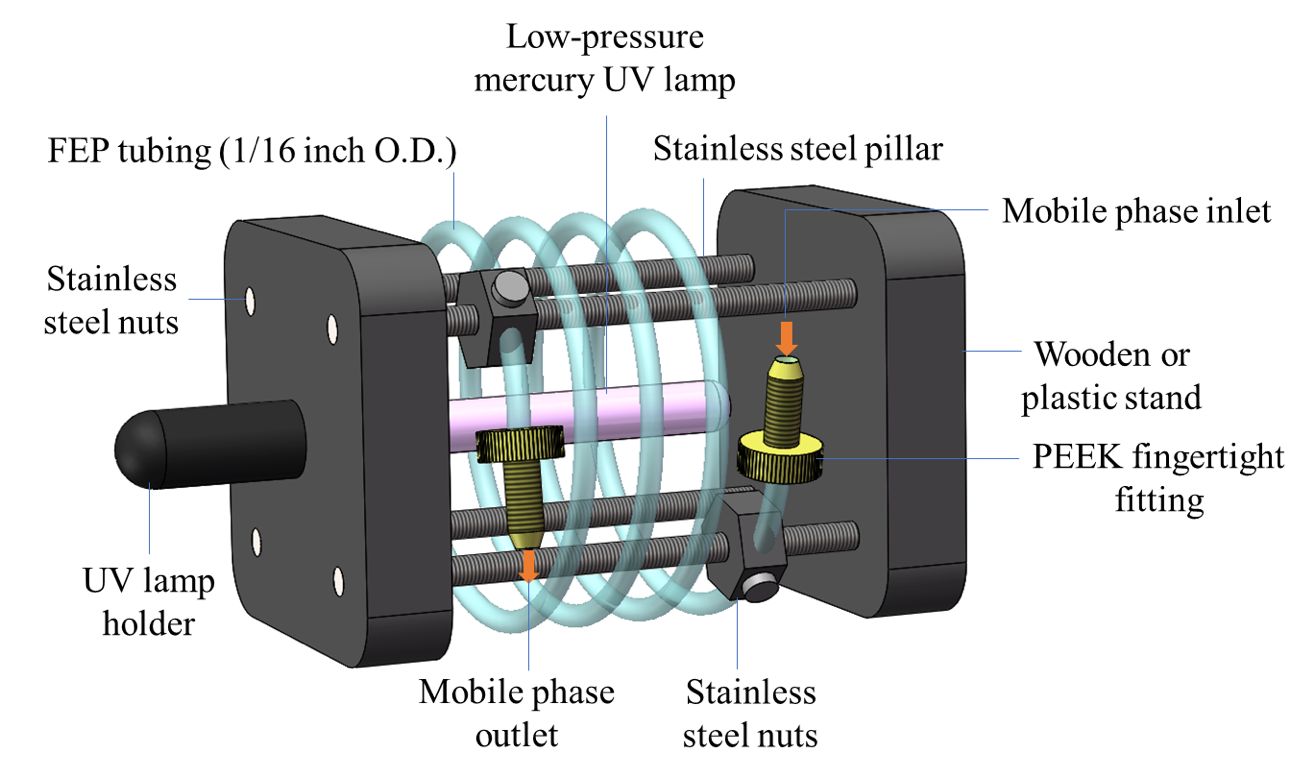


**Supplementary Figure 2.** A schematic of the flow microreactor for implementing online PB reaction in an LC-MS system.

**
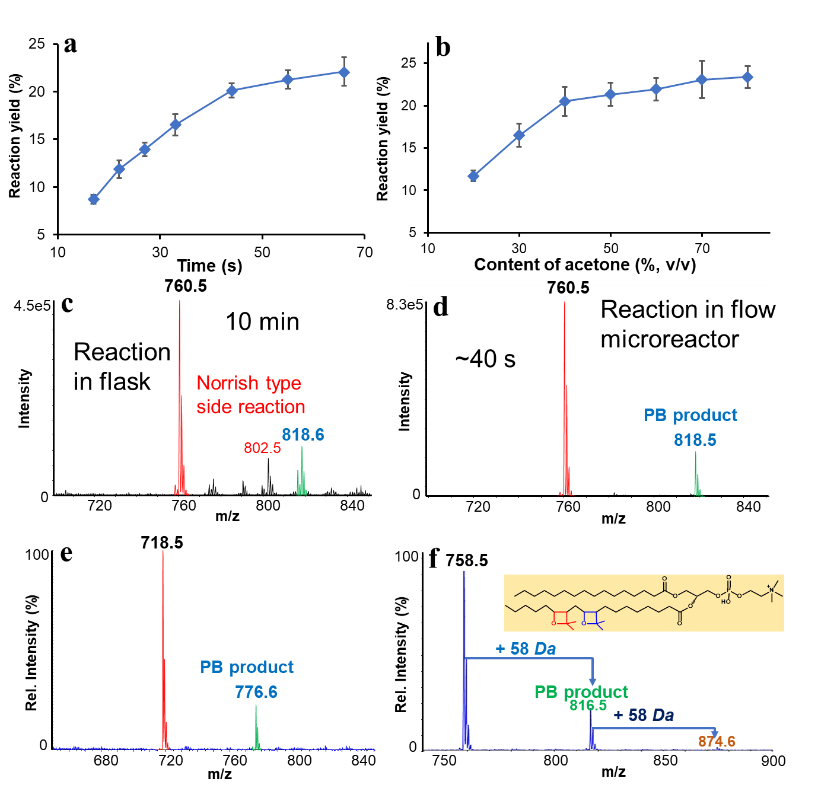
**

**Supplementary Figure 3.** The PB reaction of PC 16:0/18:1(9Z) as a function of a) the reaction time and b) the content of acetone in the solution. Comparison of positive mode ESI mass spectra of PC 16:0/18:1(9Z) after conducting the PB reaction c) in a flask for 10 min and d) through the flow microreactor for 40 s. e) Positive mode ESI mass spectrum of PE 16:0/18:1(9Z) after conducting the PB reaction in the flow microreactor. (f) Positive mode mass spectrum of PC 16:0_18:2(9Z, 12Z) by LC-PB-MS (40 s reaction). Inset shows one possible structure resulting from sequential PB reaction of PC 16:0_18:2(9Z, 12Z) due to two acetone additions.

**Supplementary Note 1. Lipid Omega Analyzer for data analysis**

In a typical PB-MS/MS spectrum, a pair of diagnostic ions at each C=C location are formed, one consisting of an aldehyde structure (F_A_) and the other with an olefin structure (F_O_). The *m/z* of these diagnostic ions can be numerically predicted as: [F_A_]=[M_PB_]-[C_ω+3_H_2(ω+3)+2-2η_] and [F_O_]=[M_PB_]-[C_ω_H_2ω+2-2η_O], where M_PB_ is the *m/z* value of PB precursor ions, ω is the same as the “ω” annotation for C=C location, while η is an integer (1, 2, 3…) that is smaller or equal to the degree of unsaturation in a single fatty acyl chain counting from the methyl end. LOA matched data from LC-PB-MS/MS with the *m/z* of the *in silico* predicted diagnostic ions under different ω and η, picked out potential diagnostic ions, determined the numerical value of (ω, η), and thus assigned C=C location of each unsaturated fatty acyl chain. For instance, two pairs of diagnostic ions, *m/z* 678/704 and 718/744 were picked out by LOA from LC-PB-MS/MS of PC 18:0_18:2 (**Supplementary Figure 4a).** Based on these values, (ω, η) were calculated as (ω=6, η=1) and (ω=9, η=2), respectively. Therefore, PC 18:0_18:2 was determined to be PC 18:0_18:2 (Δ9, 12). Similarly, the C=C location information of PE 18:1_18:2 was determined to be PE 18:1 (Δ9)_18:2 (Δ9, 12) and PE 18:1 (Δ11)_18:2 (Δ9, 12) (**Supplementary Figure 4b)**.

**
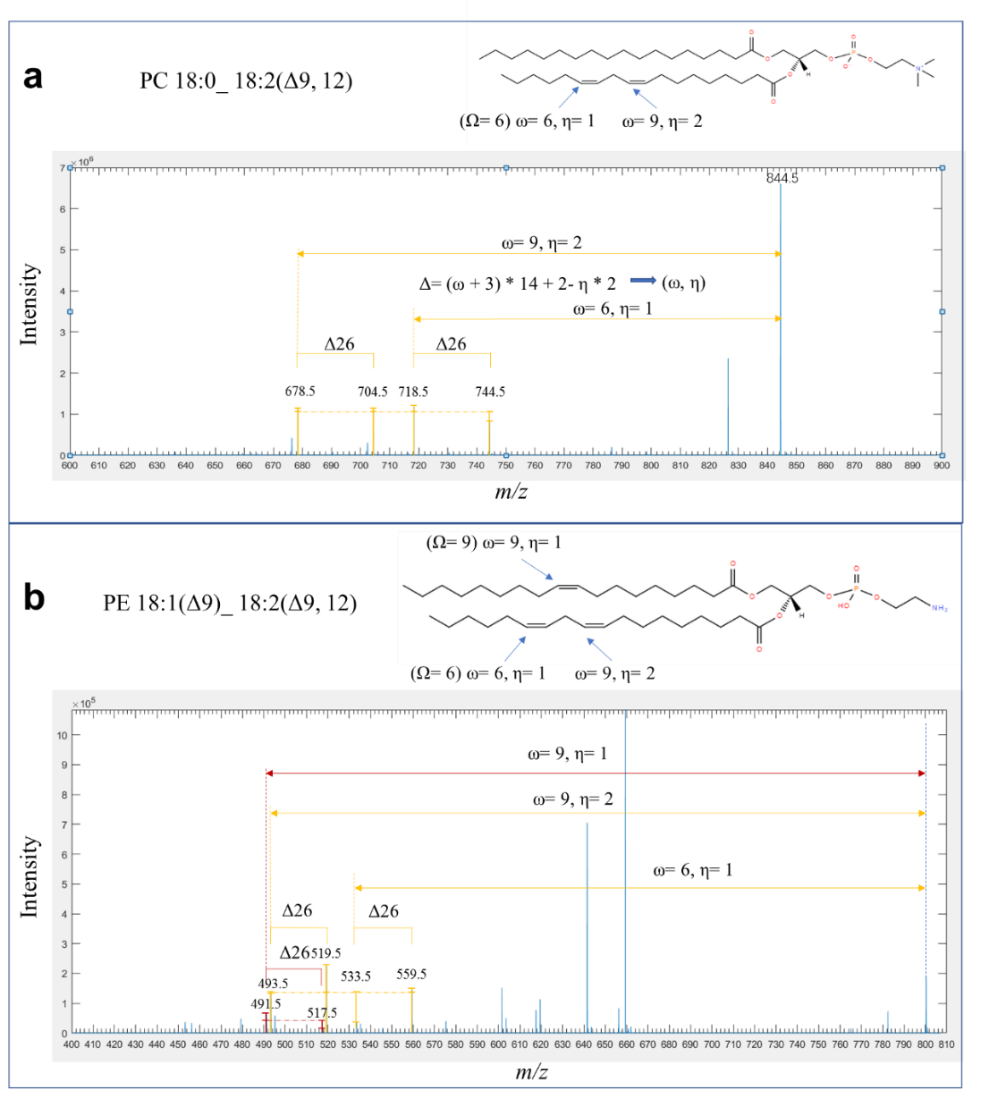
**

**Supplementary Figure 4.** Elucidation of C=C location of a) PC 18:0_18:2 and b) PE 18:1_18:2 in bovine liver extract by the Lipid Omega Analyzer.

A scoring system was developed to evaluate the accuracy of structure identification by the LOA (**Supplementary Figure 5**). The LOA first determined the number of detected diagnostic ions (A) and the theoretical number of diagnostic ions (B) for the lipid of interest (equivalent to the number of C=C multiplied by two). The value of B typically varies from 2 to 12, because for an unsaturated fatty acyl chain the number of C=C ranges from 1 to 6. LOA then normalized the ion intensities of diagnostic ions and calculated the variance (C) of intensities for diagnostic ions belonging to one single chain. Typically, the diagnostic ions produced from one unsaturated fatty acyl chain should have similar ion intensities. The score for each unsaturated fatty acyl chain can be calculated by: *Score= exp(5.87* [log(A / B) - 0.086 * C])*. When all diagnostics are identified (A/B =1) with minimum variance in ion intensities (C=0), a perfect score of 1.0 is obtained. When a majority of C=C diagnostic ions are identified (i.e. A/B chose to 1), regardless of the variation of C, a score close to 0.6 is obtained. Therefore, 0.6 is chosen as a threshold for confident identification. When only half of the theoretical diagnostic ions are identified (i.e. A/B=0.5) and the detected diagnostic ions have small variance in ion intensities (C=0), a score of 0.02 is calculated. A score smaller than 0.02 suggests suspicious identification. A score between 0.6 and 0.02, however, requires attention for manual interpretation of the data.

**Supplementary Figure 5.** Schematic of the scoring system for C=C location identification by diagnostic ions from LC-PB-MS/MS system.

For PC 18:0_18:2 (Δ9, 12) identified in bovine liver extract (data in **Supplementary Figure 4**), parameter C, the intensity variance for detected diagnostic ions, was calculated as 0.02. The number of detected diagnostic ions and the number of theoretical diagnostic ions were determined as A=4, and B=4, respectively. Based on these values, the score for the identification of chain C18:2 was calculated to as 0.98, suggesting a highly confident identification. For PE18:1 (Δ9)_18:2 (Δ9, 12), the score for the C18:1 (Δ9) and C18:2 (Δ9, 12) was 0.68 and 0.78, respectively. These scores are all larger than 0.6 and therefore suggest confident identification of the lipid structure as well.


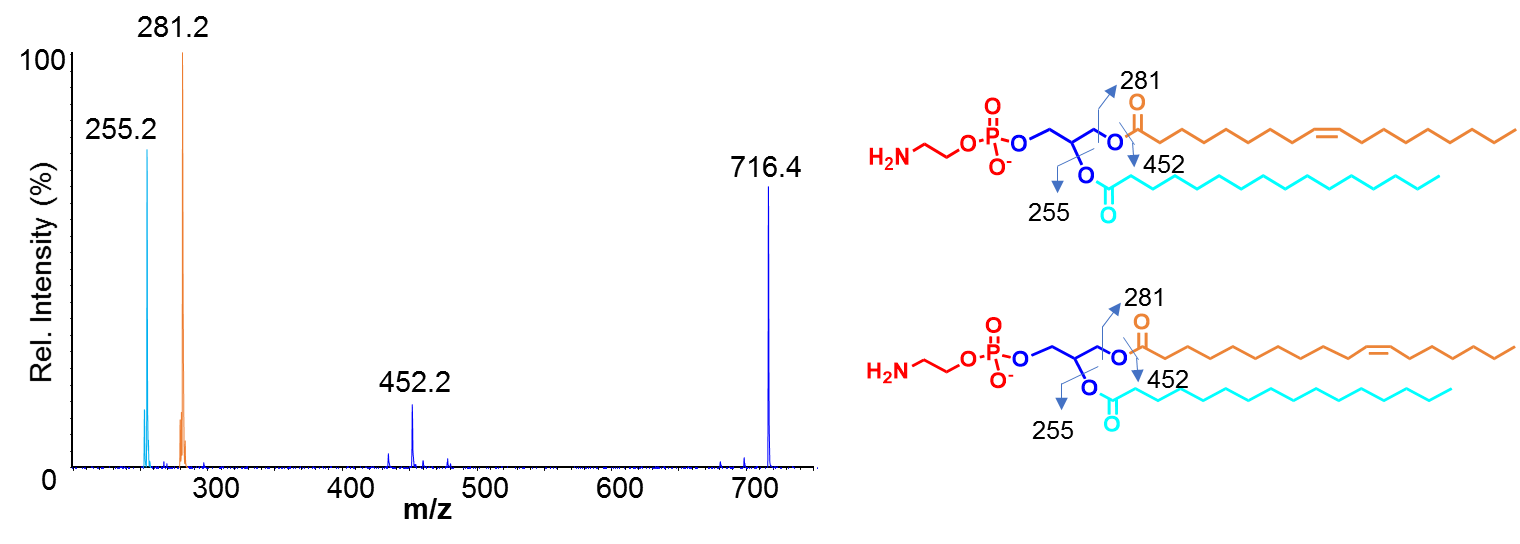


**Supplementary Figure 6.** Negative ion mode LC-MS/MS spectrum of PE 34:1(*m/z* 716.4).


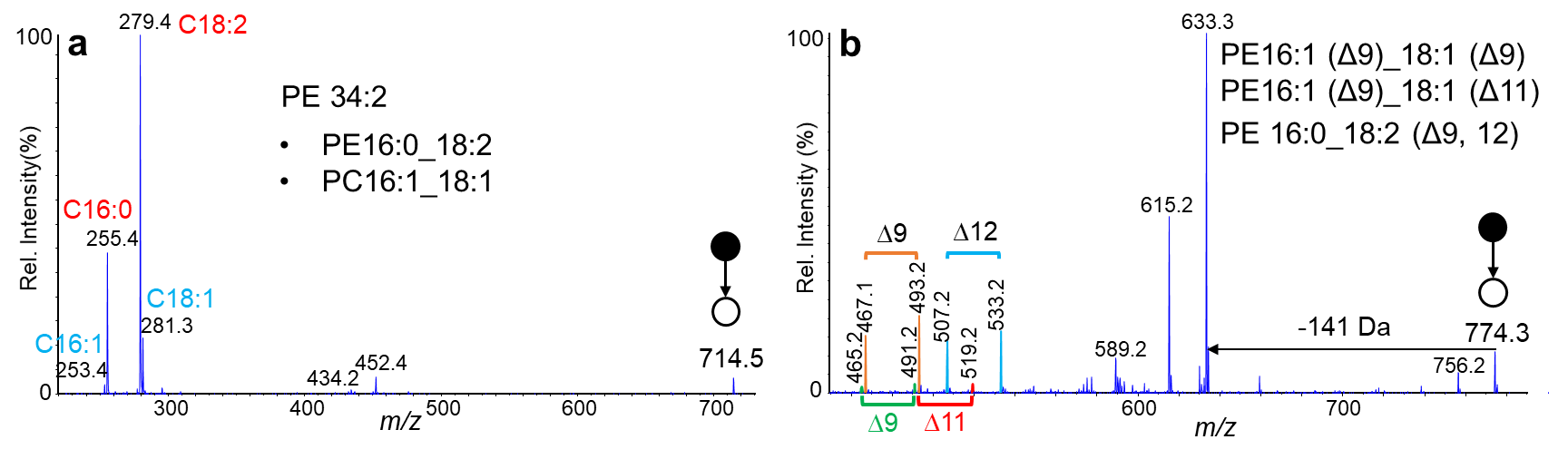


**Supplementary Figure 7.** Mass spectra of PE 34:2 in bovine liver extract by a) LC-MS/MS in negative ion mode and b) LC-PB-MS/MS in positive ion mode.


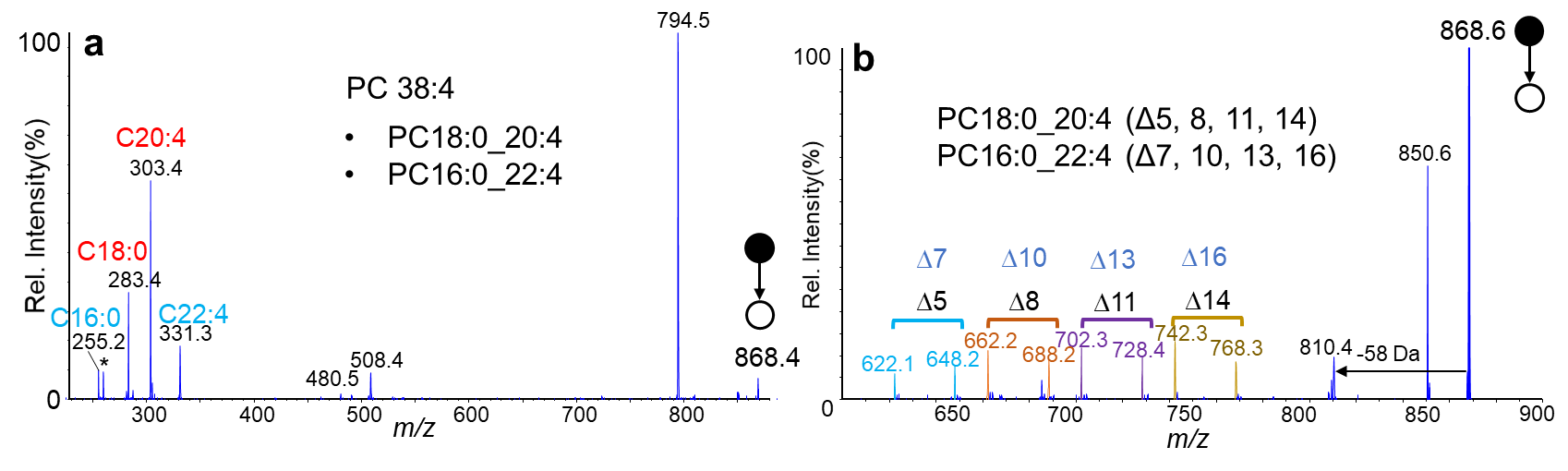


**Supplementary Figure 8.** Mass spectra of PC 38:4 in bovine liver extract by a) LC-MS/MS in negative ion mode and b) LC-PB-MS/MS in positive ion mode. * *m/z* 259, from neutral loss of 44 Da from *m/z* 303.

**
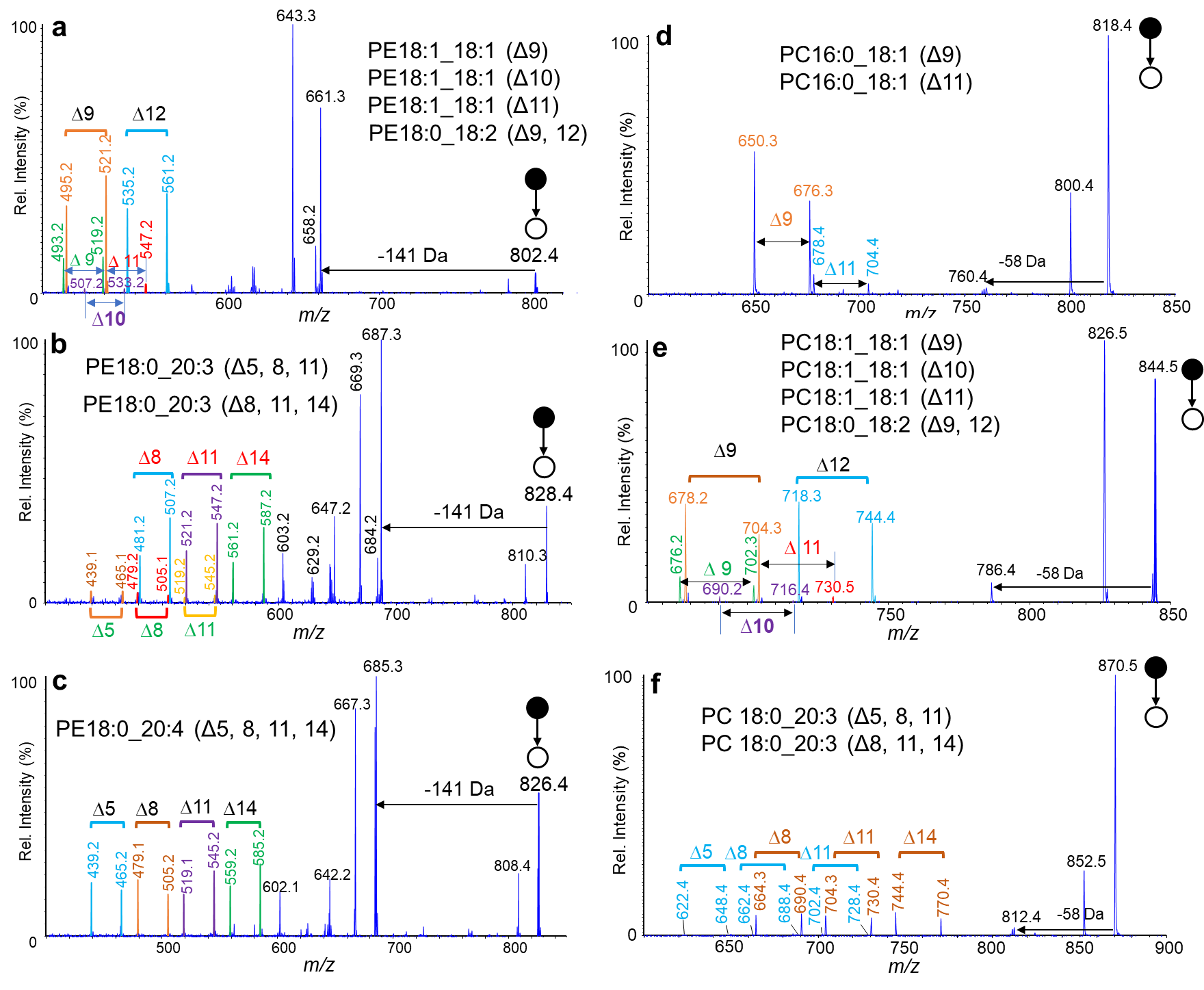
**

**Supplementary Figure 9.** Positive ion mode LC-PB-MS/MS spectra of a) PE 36:2, b) PE 38:3, c) PE 38:4, d) PC 34:1, e) PC 36:2 and f) PC38:3.


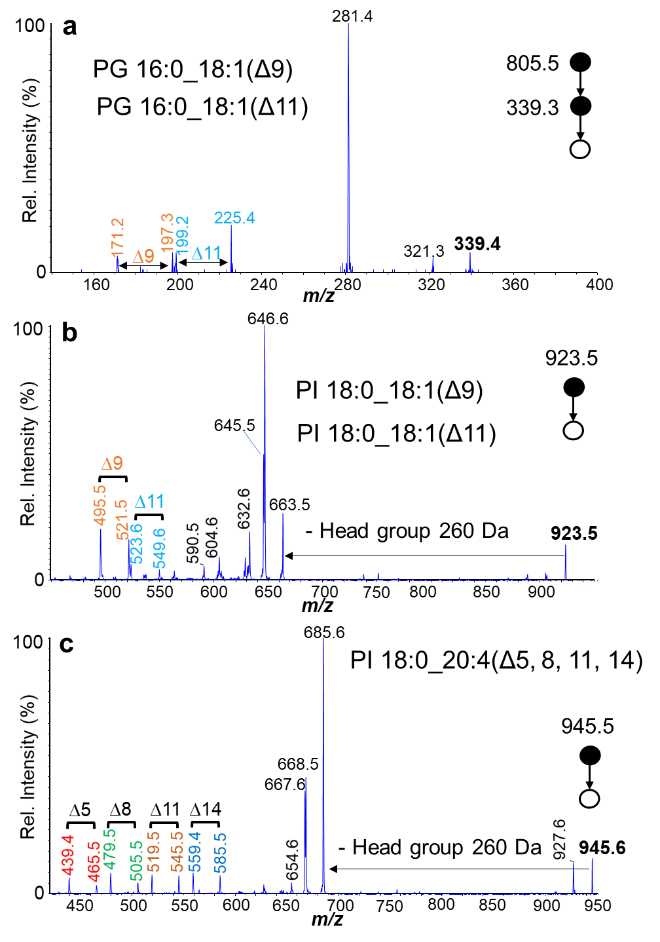


**Supplementary Figure 10.** Mass spectra of a) PG 16:0_18:1 in bovine liver extract by LC-PB-MS^3^ in negative ion mode, b) PI 36:1 and c) PI 38:4 in bovine liver extract by LC-PB-MS/MS in positive ion mode.


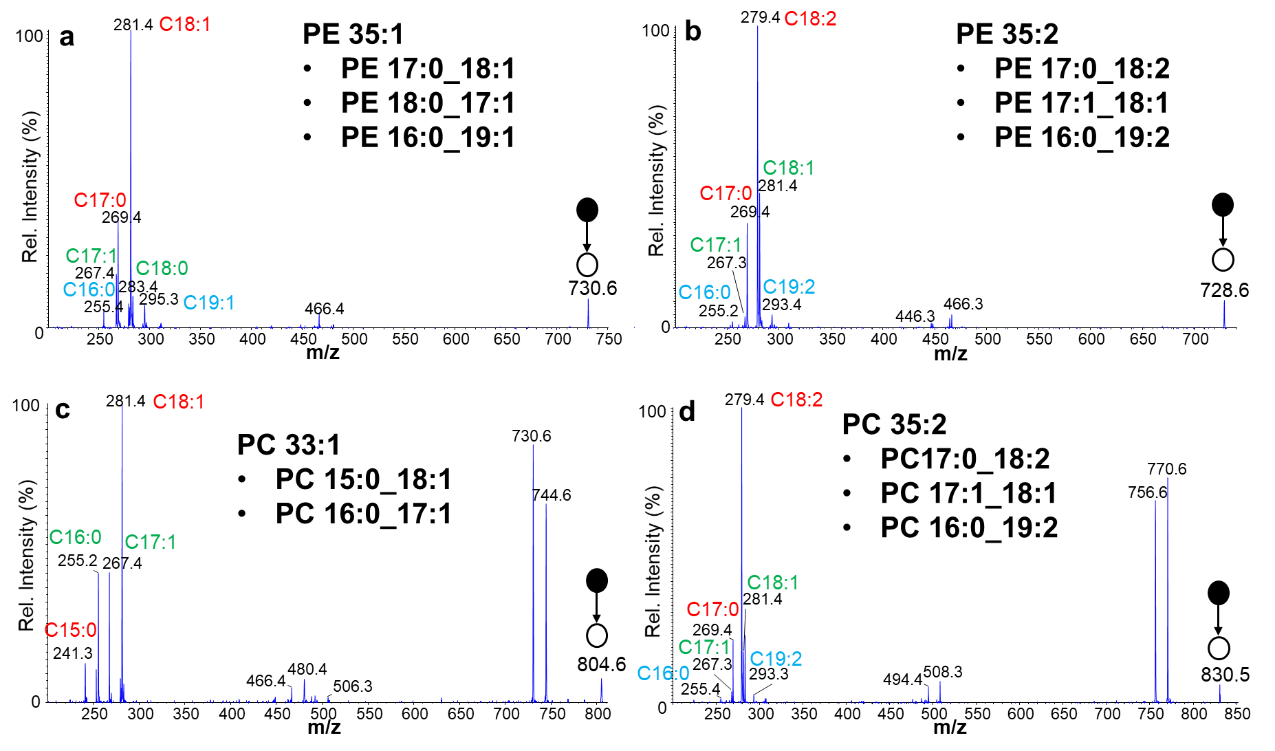


**Supplementary Figure 11.** Mass spectra of a) PE 35:1, b) 35:2, c) 33:1 and d) 35:2 in bovine liver extract by LC-MS/MS in negative ion mode.

**
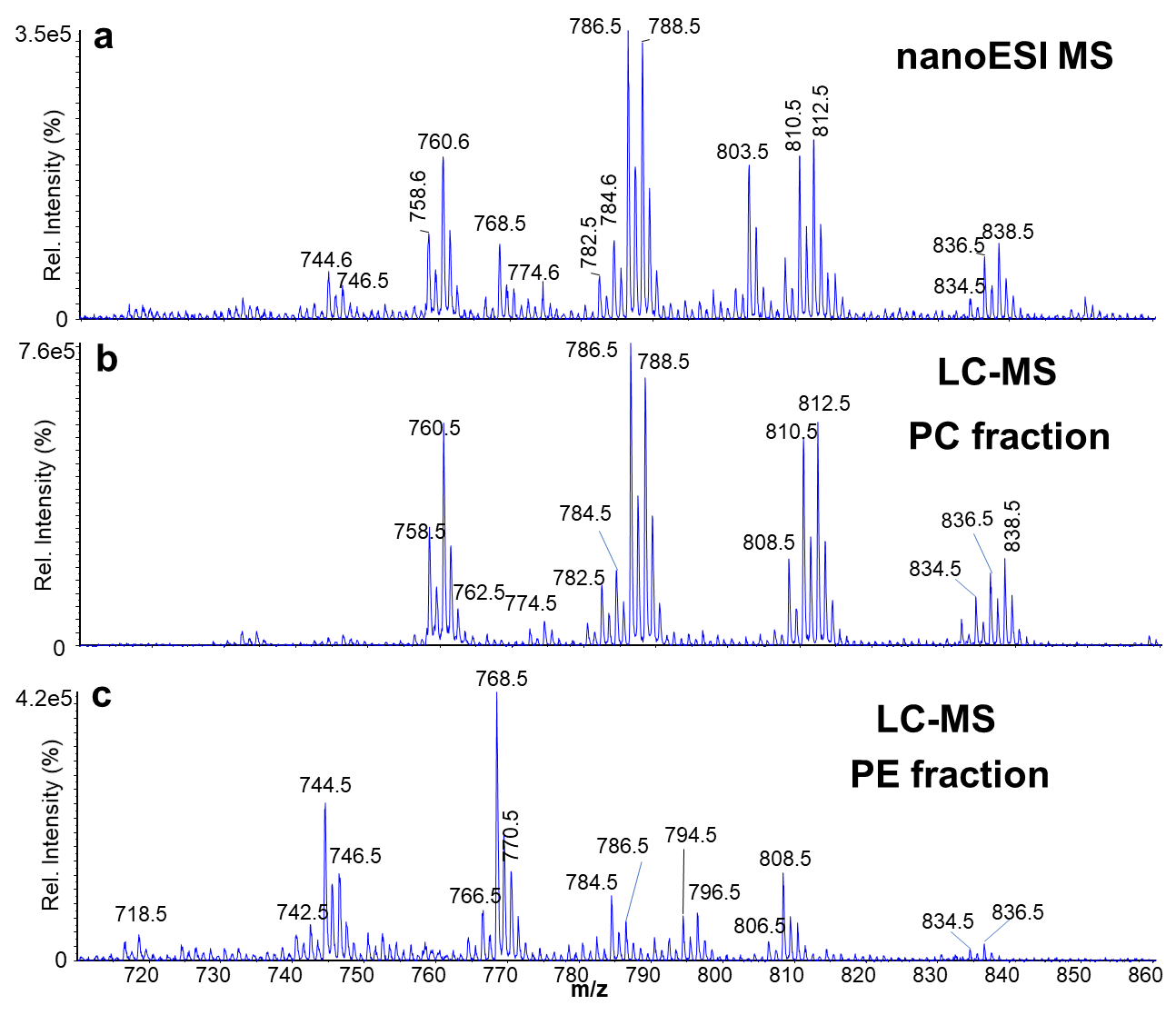
**

**Supplementary Figure 12.** Mass spectra of polar lipid extract from bovine liver, a) by nanoESI-MS, b) PC fraction by LC-MS and c) PE fraction by LC-MS. Bovine liver extract solution of 50 ppm was prepared for both nanoESI-MS and LC-MS, positive ion mode mass spectra were used for comparison.

**
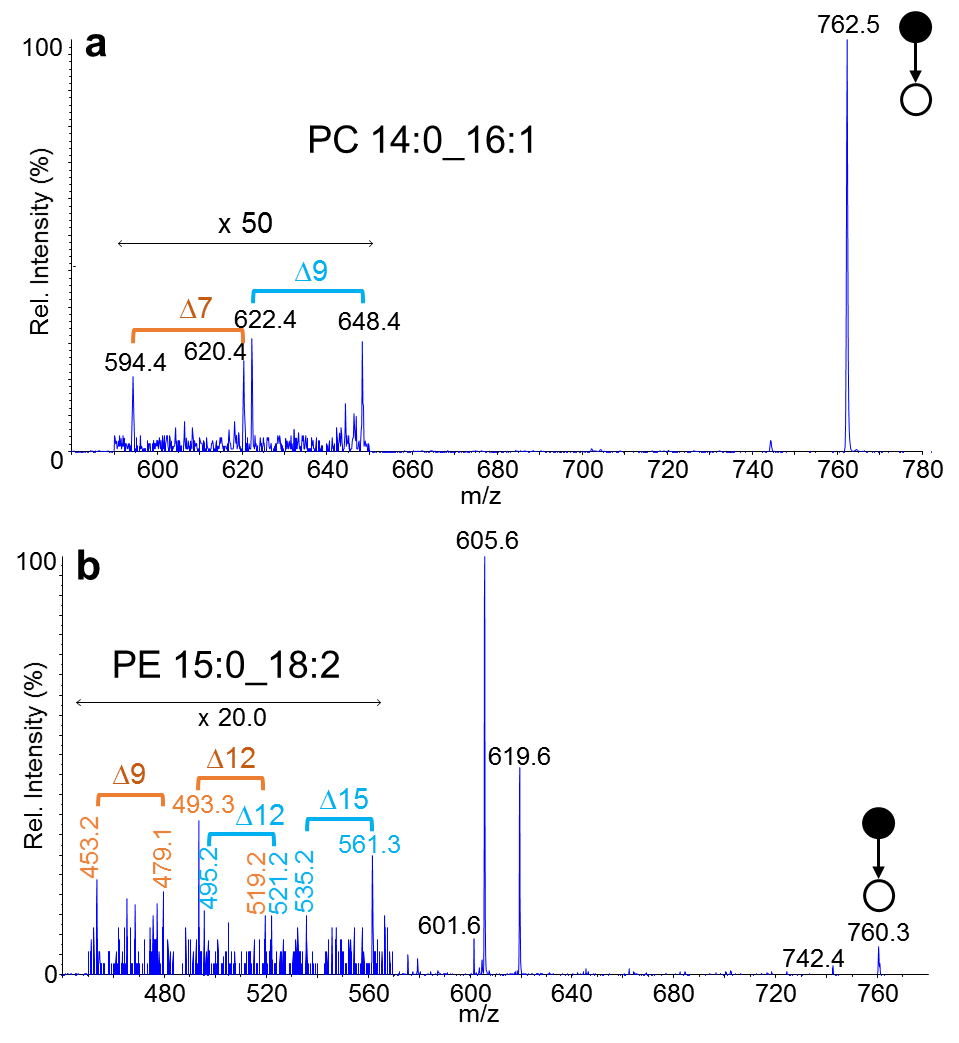
**

**Supplementary Figure 13.** Positive ion mode LC-PB-MS/MS spectra of a) PC 14:0_16:1, and b) PE 15:0_18:2 in human breast tissue samples.


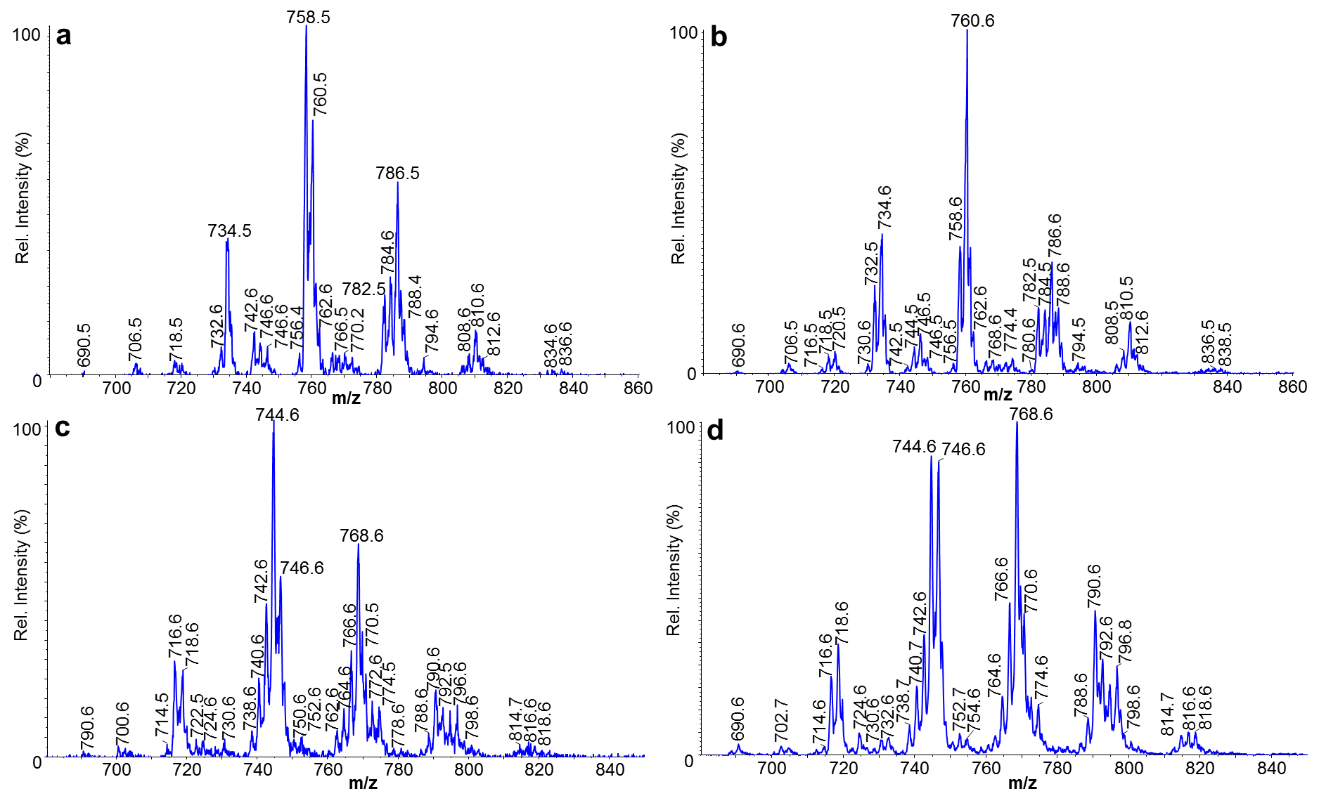


**Supplementary Figure 14.** Positive ion mode LC-MS/MS mass spectra of PC from a) normal and b) breast cancer tissue samples via PIS 184, and PE from c) normal and d) breast cancer tissue samples via NLS 141.

**
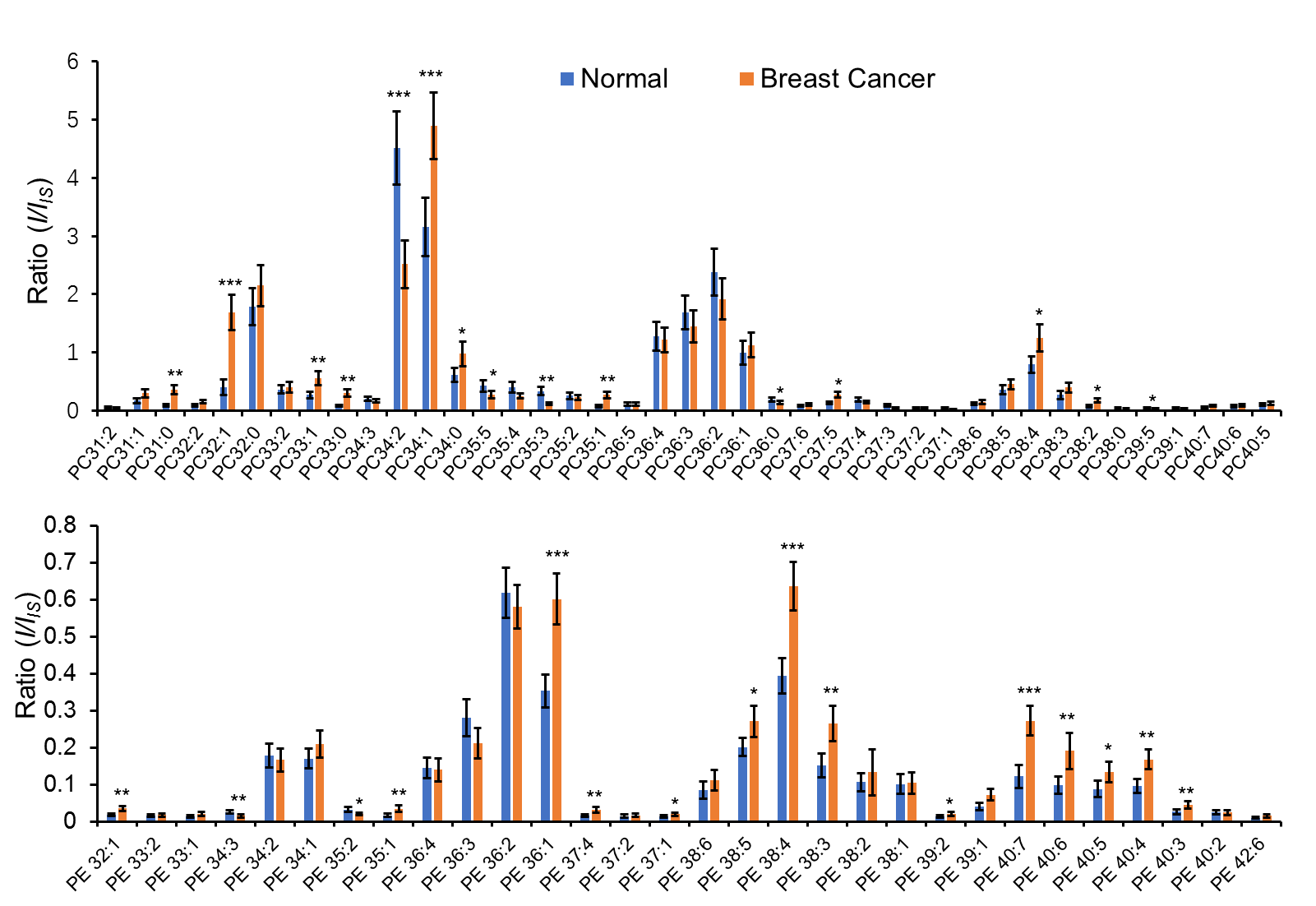
**

**Supplementary Figure 15.** Comparison of relative ion intensity of PCs and PEs in normal and cancerous breast tissue samples. PC 15:0/15:0 and PE 15:0/15:0 were used as internal standards. * P < 0.05, ** P < 0.01, *** P < 0.001 (student’s t-test). Each value represents the mean ± s.d. (N = 6).


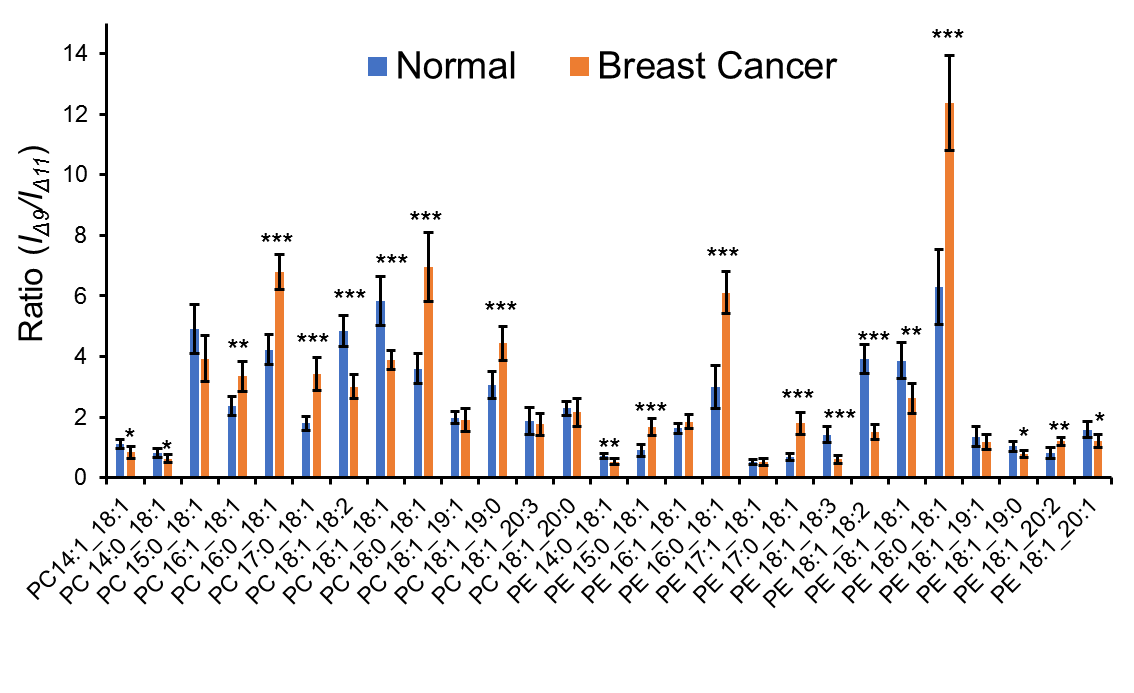


**Supplementary Figure 16.** Comparison of intensity ratio of Δ9/Δ11 C=C location isomer from C18:1 acyl chains in normal and human breast cancer tissue samples. * P < 0.05, ** P < 0.01, *** P < 0.001 (student’s t-test). Each value represents the mean ± s.d. (N = 6).


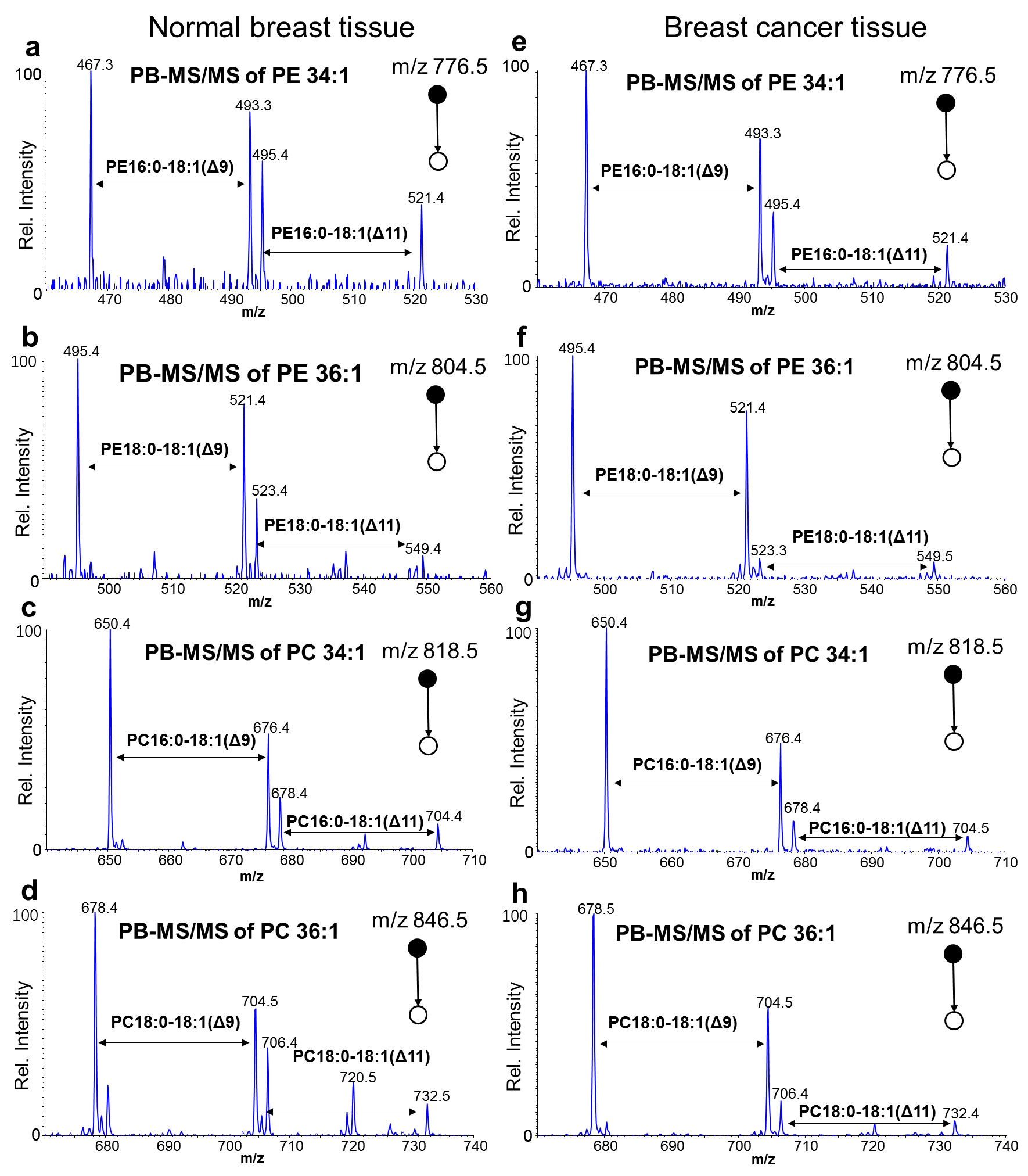


**Supplementary Figure 17.** LC-PB-MS/MS of PE 34:1 (a and e), PE 36:1 (b and f), PE 36:1 (c and g), and PE 36:1 (d and h) in normal and cancerous breast tissue samples.


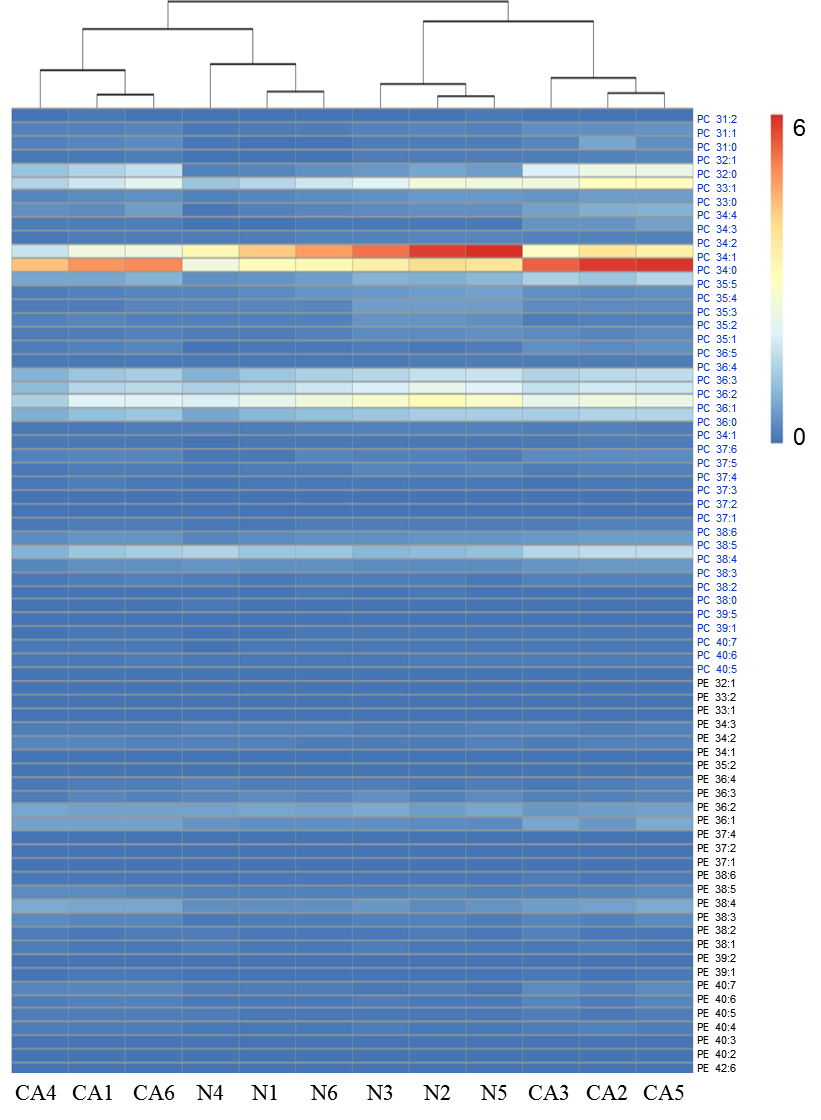


**Supplementary Figure 18.** Heatmap of relative ion intensities of PCs and PEs found in normal (N1 to N6) and cancerous (CA1 to CA6) breast tissue samples. Colors represent different relative intensities as indicated by the color bar.


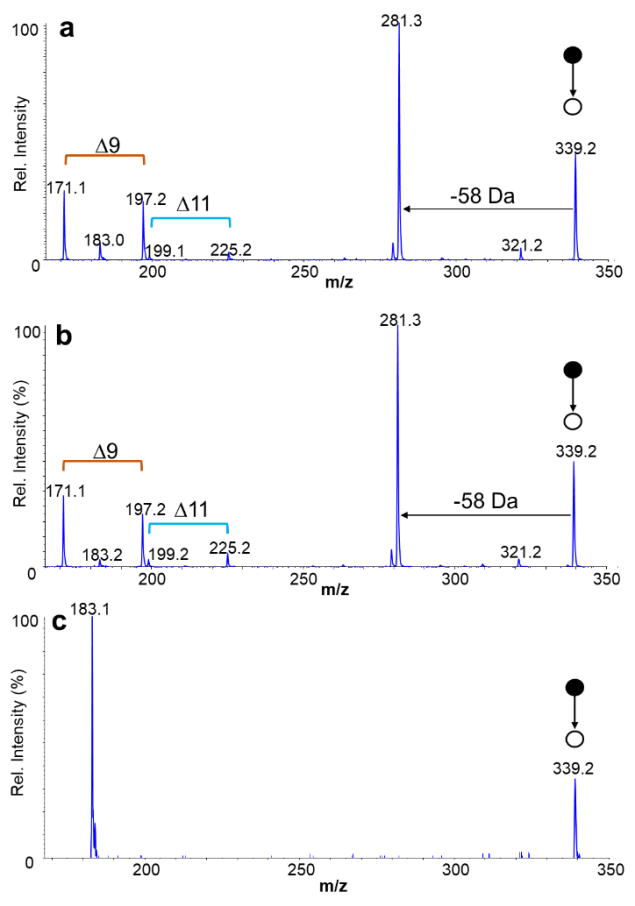


**Supplementary Figure 19.** LC-PB-MS/MS of fatty acid 18:1 in a) normal and b) cancerous breast tissue samples and c) a blank injection of methanol.


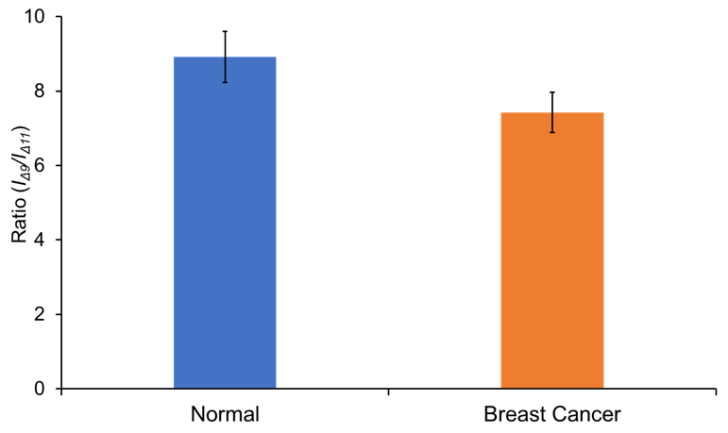


**Supplementary Figure 20.** Comparison of intensity ratio of Δ9/Δ11 C=C location isomer of FA 18:1 between normal and cancerous breast tissue samples. Each value represents the mean ± s.d. (N = 6).


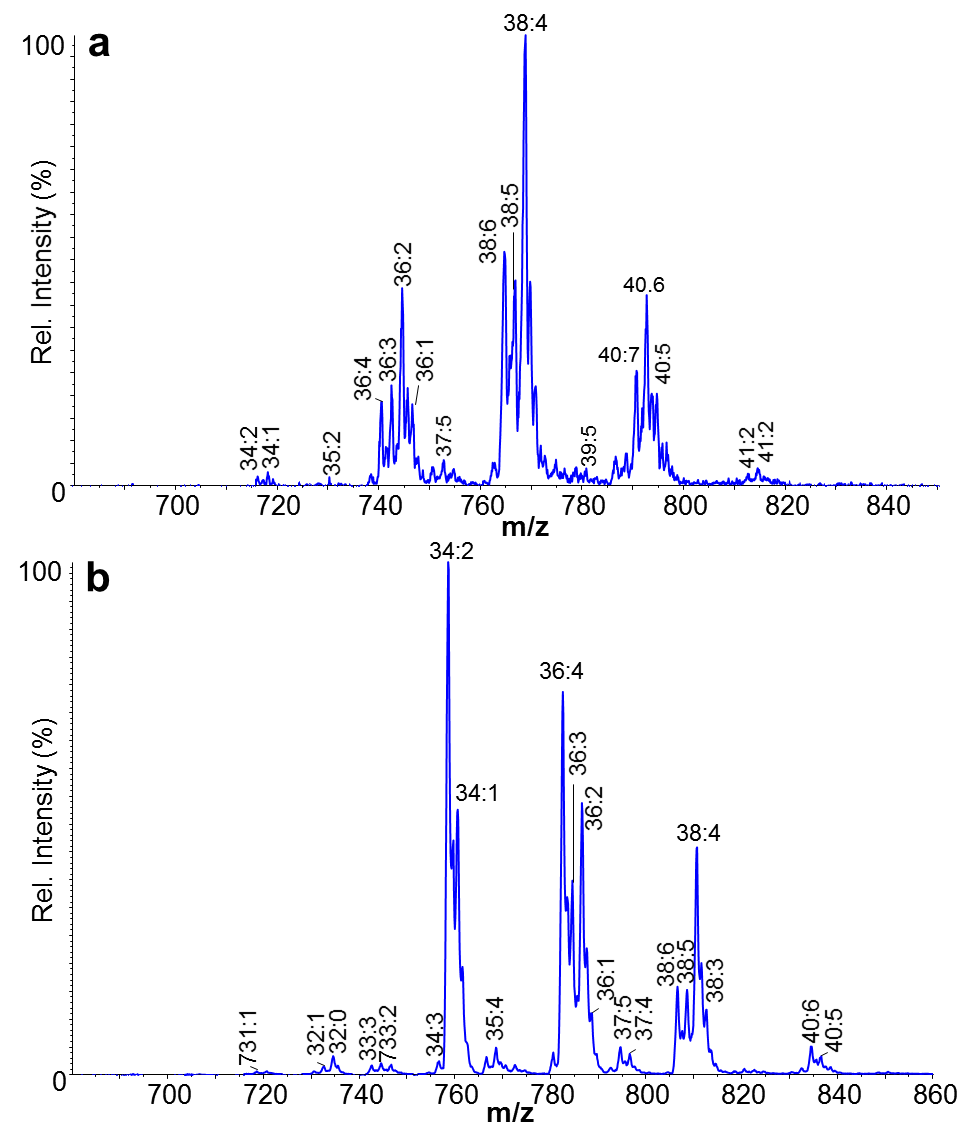


**Supplementary Figure 21.** Positive mode mass spectra of a) PC and b) PE in type 2 diabetes plasma samples.


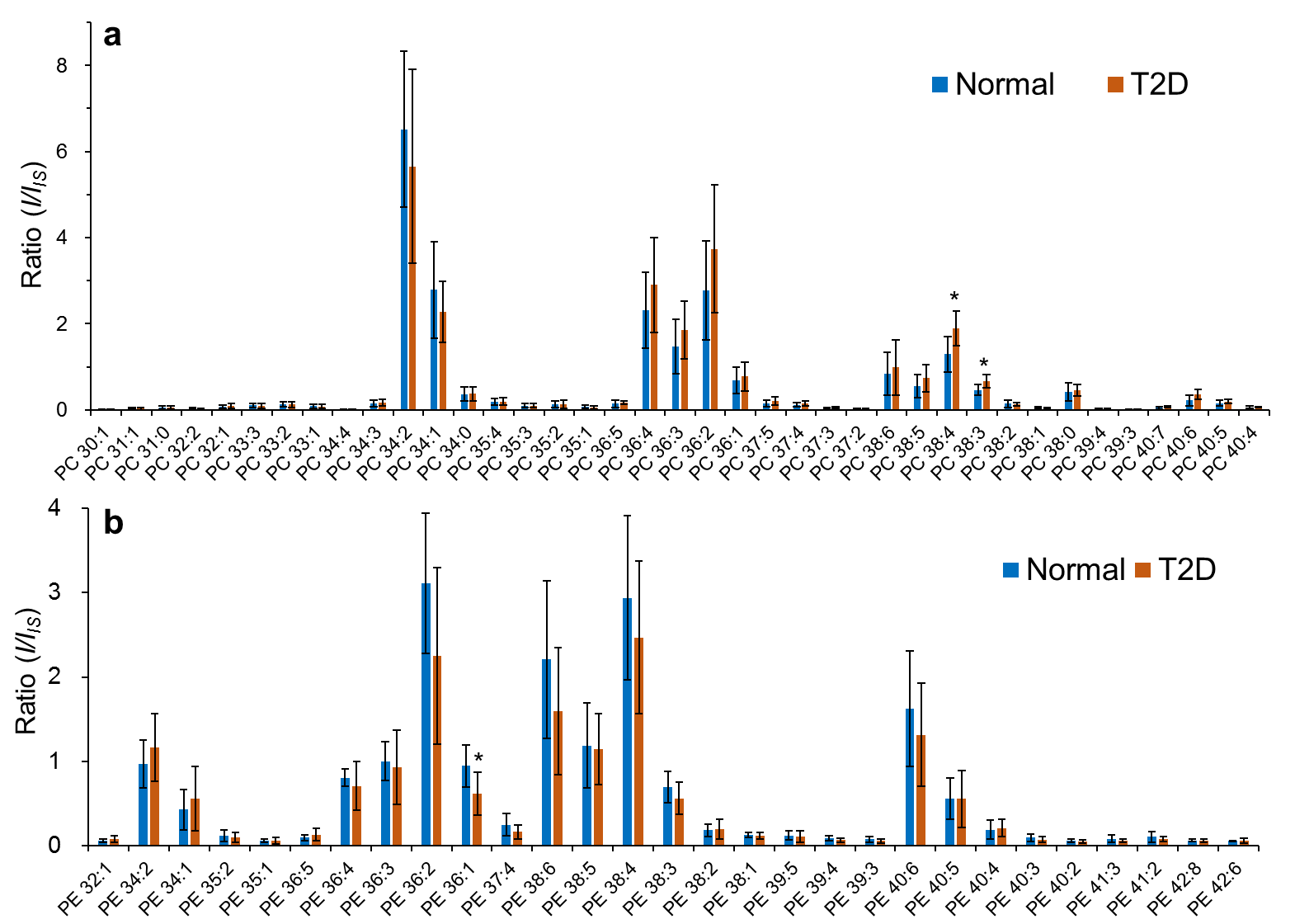


**Supplementary Figure 22.** Relative quantitation of PC and PE (I/I_IS_) by LC-MS/MS between normal and T2D plasma samples. * P < 0.05 (student’s t-test). Each value represents the mean ± s.d. (N = 6).


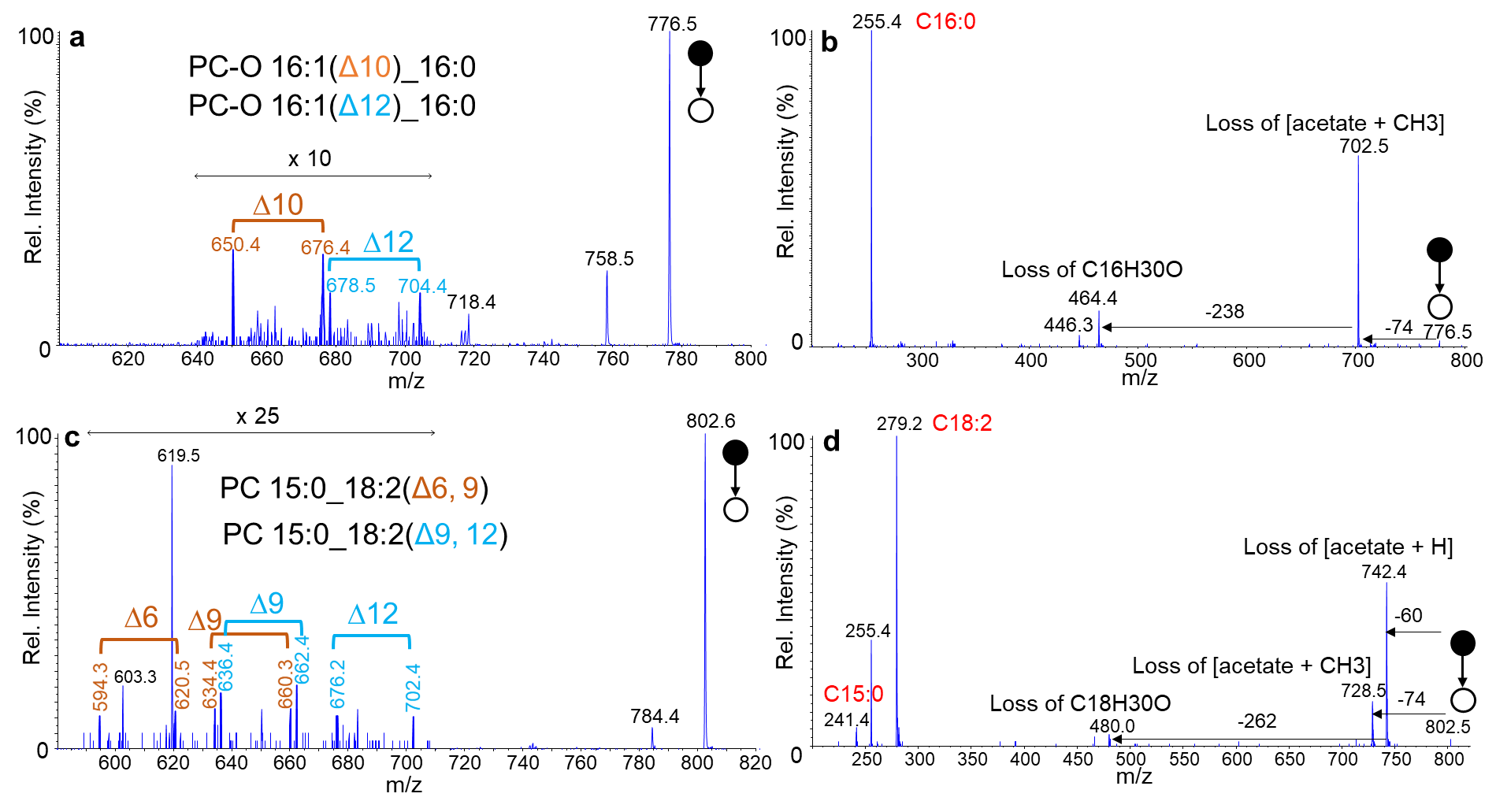


**Supplementary Figure 23.** Mass spectra of PC-O 32:1 in bovine plasma by a) LC-PB-MS/MS in positive ion mode, and b) LC-MS/MS in negative ion mode. Mass spectra of PC 33:2 in plasma by c) LC-PB-MS/MS in positive ion mode, and d) LC-MS/MS in negative ion mode.


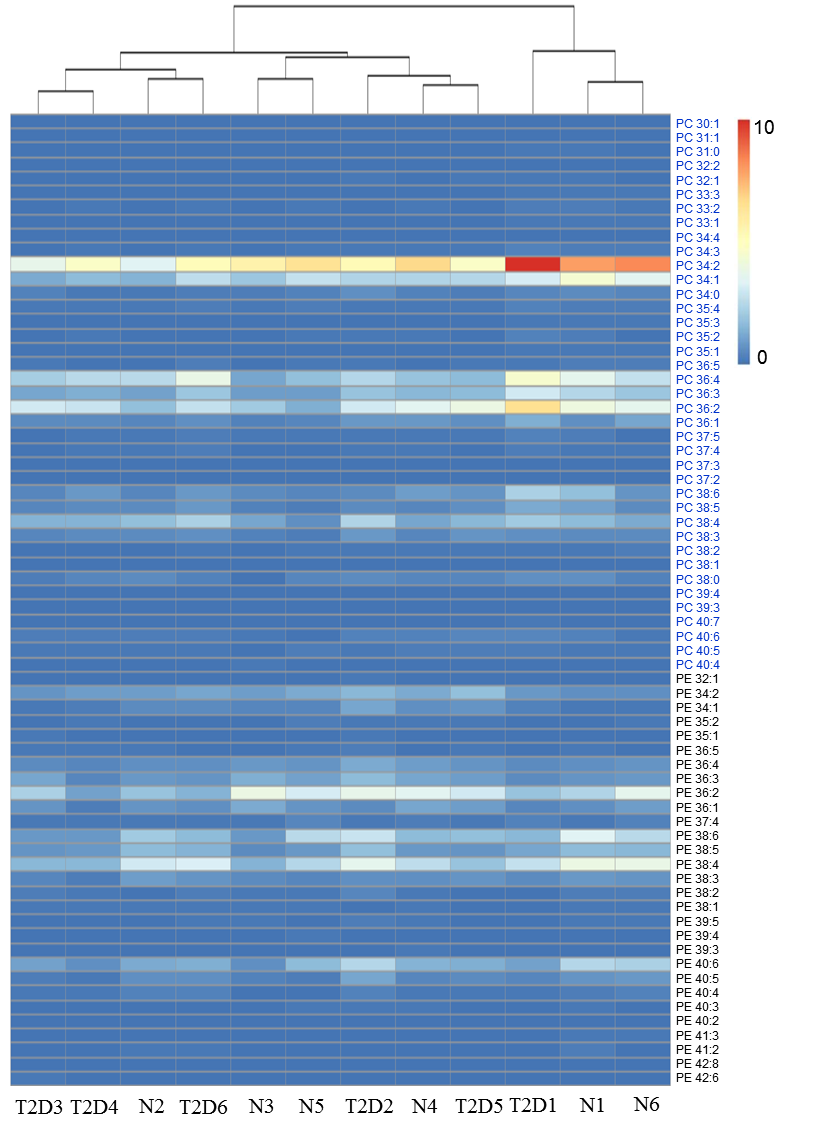


**Supplementary Figure 24.** Heatmap of relative intensities of PC and PE found in normal and T2D plasma samples. Colors represent different relative intensities as indicated by the color bar.


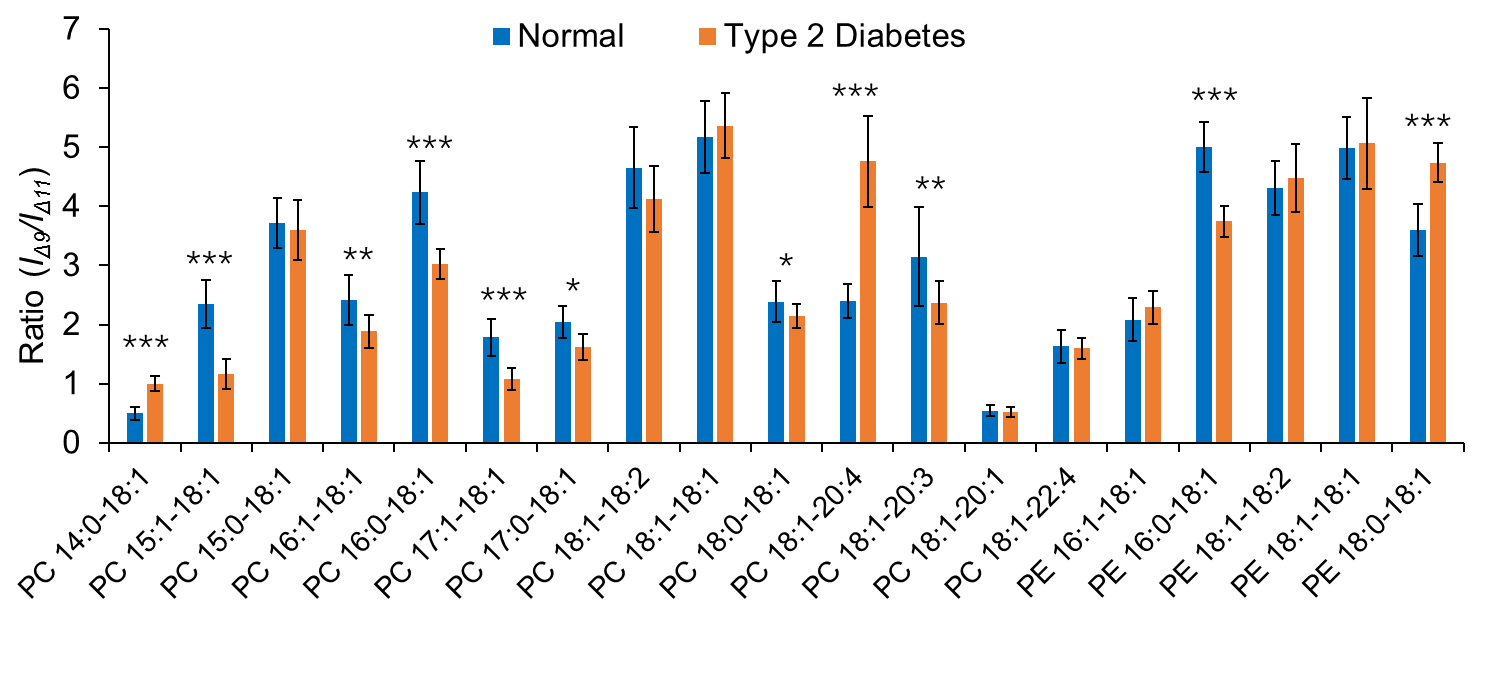


**Supplementary Figure 25.** Relative quantitation of Δ9/Δ11 C=C location isomer from C18:1 acyl chains in PC and PE of normal and T2D plasma samples. * P < 0.05, ** P < 0.01, *** P < 0.001 (student’s t-test). Each value represents the mean ± s.d. (N = 6).


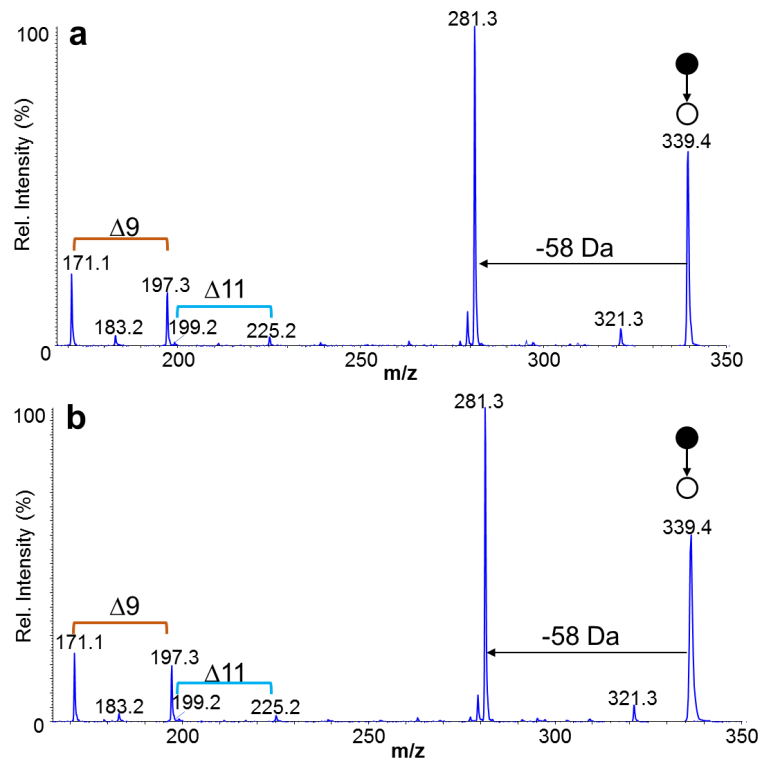


**Supplementary Figure 26.** LC-PB-MS/MS of fatty acid 18:1 in a) normal and b) T2D plasma samples.


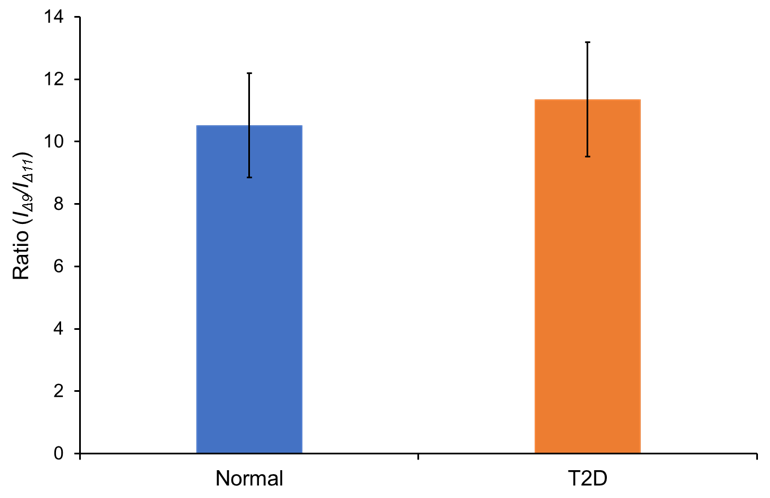


**Supplementary Figure 27.** Relative quantitation of Δ9/Δ11 C=C location isomer of FA 18:1 between normal (N = 6) and T2D plasma samples (N = 6). Each value represents the mean ± s.d.
