# Supplementary material for: Online photochemical derivatization enables comprehensive mass spectrometric analysis of unsaturated phospholipid isomers
Source: Nat Commun. 2019 Jan 8;10:79. doi: 10.1038/s41467-018-07963-8 (PMC6325166; doi:10.1038/s41467-018-07963-8)
Supplement: Supplementary file 3 — Description of Additional Supplementary Files [file 41467_2018_7963_MOESM3_ESM.docx]

**Description of Additional Supplementary Files**

**File Name**: Supplementary Data 1

**Description**: Lipid sub-class species, lipid molecular species, and lipid molecular species with C=C information from bovine liver extracts by LC-MS/MS and LC-PB-MS/MS system

**File Name:** Supplementary Data 2

**Description:** Lipid sub-class species, lipid molecular species, and lipid molecular species with C=C information from human breast cancer tissue by LC-MS/MS and LC-PB-MS/MS system.

**File Name:** Supplementary Data 3

**Description:** Lipid sub-class species, lipid molecular species, and lipid molecular species with C=C information from human plasma by LC-MS/MS and LC-PB-MS/MS system.

**File Name:** Supplementary Data 4

**Description:** Patient pathology information of breast cancer tissue samples and type 2 diabetes plasma samples.

**File Name:** Supplementary Data 5

**Description:** Mass spectrometry and LC conditions for LC-MS/MS and LC-PB-MS/MS.”
