# Supplementary material for: Online photochemical derivatization enables comprehensive mass spectrometric analysis of unsaturated phospholipid isomers
Source: Nat Commun. 2019 Jan 8;10:79. doi: 10.1038/s41467-018-07963-8 (PMC6325166; doi:10.1038/s41467-018-07963-8)
Supplement: Supplementary file 9 — Reporting Summary [file 41467_2018_7963_MOESM9_ESM.pdf]

nature research

Corresponding author(s): Zheng Ouyang
Yu Xia

Reporting Summary

Nature Research wishes to improve the reproducibility of the work that we publish. This form provides structure for consistency and transparency in reporting. For further information on Nature Research policies, see Authors & Referees and the Editorial Policy Checklist.

Statistical parameters

When statistical analyses are reported, confirm that the following items are present in the relevant location (e.g. figure legend, table legend, main text, or Methods section).

n/a	Confirmed	
☐	☒	The exact sample size (n) for each experimental group/condition, given as a discrete number and unit of measurement
☐	☒	An indication of whether measurements were taken from distinct samples or whether the same sample was measured repeatedly
☐	☒	The statistical test(s) used AND whether they are one- or two-sided<br>*Only common tests should be described solely by name; describe more complex techniques in the Methods section.*
☒	☐	A description of all covariates tested
☒	☐	A description of any assumptions or corrections, such as tests of normality and adjustment for multiple comparisons
☐	☒	A full description of the statistics including central tendency (e.g. means) or other basic estimates (e.g. regression coefficient) AND variation (e.g. standard deviation) or associated estimates of uncertainty (e.g. confidence intervals)
☐	☒	For null hypothesis testing, the test statistic (e.g. F, t, r) with confidence intervals, effect sizes, degrees of freedom and P value noted<br>*Give P values as exact values whenever suitable.*
☒	☐	For Bayesian analysis, information on the choice of priors and Markov chain Monte Carlo settings
☐	☒	For hierarchical and complex designs, identification of the appropriate level for tests and full reporting of outcomes
☒	☐	Estimates of effect sizes (e.g. Cohen's d, Pearson's r), indicating how they were calculated
☐	☒	Clearly defined error bars<br>*State explicitly what error bars represent (e.g. SD, SE, CI)*

Our web collection on statistics for biologists may be useful.

Software and code

Policy information about availability of computer code

Data collection	Analyst 1.6.3 from Sciex
Data analysis	Analyst 1.6.3 from Sciex, R 3.4.1, Lipid Omega Analyzer (LOA, available at https://github.com/LipidAnalysis?tab=repositories)

For manuscripts utilizing custom algorithms or software that are central to the research but not yet described in published literature, software must be made available to editors/reviewers upon request. We strongly encourage code deposition in a community repository (e.g. GitHub). See the Nature Research guidelines for submitting code & software for further information.

Data

Policy information about availability of data

All manuscripts must include a data availability statement. This statement should provide the following information, where applicable:

- Accession codes, unique identifiers, or web links for publicly available datasets
- A list of figures that have associated raw data
- A description of any restrictions on data availability

The LOA software and representative MGF input files are available on Github (https://github.com/LipidAnalysis?tab=repositories). The code of the LOA is available from the corresponding authors upon reasonable request.

Data generated or analysed during this study are included in this article its supplementary information files.
Figure 2, Figure 3 and Figure 4 have associated excel files in the supplementary information.
These are no restrictions on data availability.

Field-specific reporting

Please select the best fit for your research. If you are not sure, read the appropriate sections before making your selection.

☒ Life sciences ☐ Behavioural & social sciences ☐ Ecological, evolutionary & environmental sciences

For a reference copy of the document with all sections, see nature.com/authors/policies/ReportingSummary-flat.pdf

Life sciences study design

All studies must disclose on these points even when the disclosure is negative.

Sample size	We used 6 breast cancer tissues, 6 normal plasma samples and 6 type 2 diabetes plasma samples. These samples were just used to demonstrate the possible application of the developed lipidomic approach, but not for discover the actual biomarkers. Sample size of 6 was show a relative reproducibility of the lipid analysis results.
Data exclusions	No data were excluded.
Replication	Experimental findings were reliably reproduced.
Randomization	Samples were grouped into normal/breast cancer tissue samples, and normal/type 2 diabetes samples according to the diagnosis results. No further grouping was applied.
Blinding	Blinding was not applied, because the demonstrations in normal/breast cancer tissue samples, and normal/type 2 diabetes samples were just to show the difference between two groups of samples.

Reporting for specific materials, systems and methods

Materials & experimental systems

n/a	Involved in the study	
☒	☐	Unique biological materials
☒	☐	Antibodies
☒	☐	Eukaryotic cell lines
☒	☐	Palaeontology
☒	☐	Animals and other organisms
☐	☒	Human research participants

Methods

n/a	Involved in the study	
☒	☐	ChIP-seq
☒	☐	Flow cytometry
☒	☐	MRI-based neuroimaging

Human research participants

Policy information about studies involving human research participants

Population characteristics	Samples were obtained from specimen bank of the hospital. All the samples were from Asian individuals, breast cancer tissue samples were from women patients at the age of 38-64. Normal plasma were from healthy individuals at the age of 41-65, with blood fasting glucose concentration < 6.1 mmol/L. Type 2 diabetes plasma were from individuals at the age of 42-78, with blood fasting glucose concentration > 7 mmol/L, without significant complex diseases.
Recruitment	No relevant. Samples were obtained from specimen bank of the hospital.
